# Supplementary material for: Correlation between Expression Profiles of Key Signaling Genes in Colorectal Cancer Samples from Type 2 Diabetic and Non-Diabetic Patients
Source: Life (Basel). 2020 Sep 22;10(9):216. doi: 10.3390/life10090216 (PMC7555724; doi:10.3390/life10090216)
Supplement: Supplementary file 1 [file life-10-00216-s001.zip › life-892598-supplementary- for XML/Supplementary-Figure S1 and S2.docx]

**Figure S1.** A heat map featuring transcript levels with unsupervised
clustering showing the expression levels of each gene for each sample taken.

Expression levels of all transcripts successfully assayed on the OpenArray panel are shown for all patient pools. Missing data spots are marked by black boxes. Rows represent gene transcripts (gene expression measurement assays), while columns correspond to a total of 12 sample pools. Clustering has been performed by the Euclidean method applying average linkage. Pool codes are shown at the bottom of the panel: C1-3: healthy controls, T1-3: CRC patients, D1-3: T2D patients, DT1-3: CRC + T2D patients. Where two different assays for the same gene were included in the OpenArray panel, assays are distinguished by an underscore followed by a letter-figure combination. The corresponding assays are as follows: BAX_1: Hs00180269_m1, BAX_2: Hs01016552_g1, BCL2_1: Hs00608023_m1, BCL2_2: Hs99999018_m1, CCL20_1: Hs00355476_m1, CCL20_2: Hs01011368_m1, CYP19A1_1: Hs00903411_m1, CYP19A1_2: Hs00903413_m1, FAS_1: Hs00236330_m1, FAS_2: Hs00531110_m1, FASN_1: Hs00188012_m1, FASN_2: Hs01005622_m1, IL8_1: Hs00174103_m1, IL8_2: Hs99999034_m1, JUN_1: Hs00277190_s1, JUN_2: Hs99999141_s1, LEF_1: Hs00212390_m1, LEF_2: Hs01547250_m1, LTA_1: Hs00236874_m1, LTA_2: Hs99999086_m1, MDM2_1: Hs00234753_m1, MDM2_2: Hs01066938_m1, MYC_1: Hs00905030_m1, MYC_2: Hs99999003_m1, NAIP_1: Hs01847653_s1, NAIP_2: Hs03037952_m1, PRKCA_1: Hs00176973_m1, PRKCA_2: Hs00925195_m1, RBP1_1: Hs01011512_g1, RBP1_2: Hs01011514_m1, SELE_1: Hs00174057_m1, SELE_2: Hs00950401_m1, TERT_1: Hs00972650_m1, TERT_2: Hs00972656_m1

**Figure 2.** A heat map of the 23 assayed by qPCR.

Expression levels of 23 individually assayed transcripts are classified for both patient groups and tissue types. Missing data spots are marked by black boxes. Rows represent genes, columns represent samples. Clustering has been carried out by the Euclidean method applying average linkage. Codes at the bottom of the panel identify samples as follows: ND_T1-6: non-diabetes tumor tissue, ND_S1-6: non-diabetes surrounding tissue, D_T1-6: diabetes tumor tissue, D_S1-6: diabetes surrounding tissue
