# Supplementary material for: Correlation between Expression Profiles of Key Signaling Genes in Colorectal Cancer Samples from Type 2 Diabetic and Non-Diabetic Patients
Source: Life (Basel). 2020 Sep 22;10(9):216. doi: 10.3390/life10090216 (PMC7555724; doi:10.3390/life10090216)
Supplement: Supplementary file 1 [file life-10-00216-s001.zip › life-892598-supplementary- for XML/Supplementary-Tables S1 and S2.docx]

Results from the colonoscopy samples assayed on the OpenArray™ Human Signal Transduction Panel are shown. Only transcripts showing significantly different expression levels between tumor versus non-tumor (panel A) or between diabetic versus non-diabetic subjects (panel B) are indicated (p ≤ 0.05 by 2-way ANOVA). Genes whose expression was interrogated with more than one TaqMan^®^ assays (with different primers and probes) on the panel are shown in italics. In these cases, precise assay identifiers are indicated with upper index figures.

**Table S1.** Transcripts with significantly different expression levels either by tumor or by diabetes status.

Panel A

| **Gene** | **p Value  (Tumor vs. Non-Tumor)** | **Fold Change in Tumor*** | **Gene** | **p Value  (Tumor vs. Non-Tumor)** | **Fold Change in Tumor*** |
| --- | --- | --- | --- | --- | --- |
| **ADCY5** | 0.0003 | 0.33 | **TGFB2** | 0.0207 | 2.85 |
| **PRDX6** | 0.0005 | 0.48 | **CDK4** | 0.0221 | 2.15 |
| **CDKN2B** | 0.0008 | 0.11 | **FZD1** | 0.0221 | 0.63 |
| **NPY** | 0.0008 | 0.17 | **KRAS** | 0.0221 | 0.48 |
| **ODC1** | 0.0008 | 2.08 | **NFKB2** | 0.0222 | 1.53 |
| **PDGFB** | 0.0009 | 1.87 | **PRKRA** | 0.0224 | 1.45 |
| **BGLAP** | 0.0015 | 0.20 | ***BAX^3^*** | *0.0233* | *1.37* |
| ***FASN^1^*** | *0.0015* | *1.73* | **CRHR2** | 0.0249 | 0.38 |
| **MAPK3** | 0.0015 | 0.57 | ***GAPDH^4^*** | *0.0252* | *1.70* |
| **RIPK2** | 0.0018 | 2.38 | **SSTR2** | 0.0252 | 0.23 |
| **WNT2** | 0.0033 | 48.49 | **BMP3** | 0.0268 | 0.19 |
| **CCNE1** | 0.0034 | 1.98 | ***MYC^5^*** | *0.027* | *2.46* |
| **FZD5^#^** | 0.0035 | 0.52 | ***MYC^6^*** | *0.0272* | *2.68* |
| **CYR61** | 0.0036 | 4.82 | **ARRB1** | 0.0277 | 0.49 |
| **TLR7** | 0.0038 | 0.40 | **CALM1^#^** | 0.0282 | 0.83 |
| **BMP2** | 0.0057 | 0.53 | **PTH1R** | 0.0299 | 0.56 |
| **SOCS2** | 0.006 | 0.64 | **FZD3** | 0.0301 | 1.86 |
| **FOXA2^#^** | 0.0061 | 1.82 | **PTCH2** | 0.0313 | 0.62 |
| **ITGB7** | 0.0061 | 0.47 | **CXXC4** | 0.0315 | 2.67 |
| **CCNA2** | 0.0065 | 1.71 | **SOD2** | 0.0315 | 2.10 |
| **RHOU** | 0.007 | 0.62 | **MMP3** | 0.0317 | 25.24 |
| **TGFB3** | 0.0071 | 1.79 | **GRB10^#^** | 0.0324 | 1.60 |
| **MAPK8IP2** | 0.0077 | 0.25 | **TNFRSF10B^#^** | 0.0325 | 1.78 |
| **BRCA1** | 0.0087 | 1.99 | **EDN1** | 0.0327 | 0.73 |
| **GYS1** | 0.0088 | 0.63 | **NR4A2** | 0.0333 | 3.24 |
| **GIPR** | 0.0089 | 0.31 | **GRM7** | 0.0334 | 0.38 |
| **MMP9** | 0.0094 | 4.27 | **IL10RA** | 0.0349 | 0.57 |
| **BMP7** | 0.0097 | 4.93 | **CDX1** | 0.0353 | 0.54 |
| **PDK2** | 0.0105 | 0.55 | **PECAM1** | 0.0361 | 1.85 |
| **TGFBI** | 0.0105 | 4.64 | **HMOX1** | 0.0374 | 0.65 |
| **RPS6KA1** | 0.0107 | 0.60 | **MAPK7** | 0.0392 | 0.71 |
| **IL6** | 0.0111 | 24.78 | ***LEF1^7^*** | *0.0393* | *4.20* |
| **RHEB** | 0.0112 | 2.02 | **SARM1** | 0.0406 | 0.70 |
| **INHBA** | 0.0123 | 18.95 | **BIRC5** | 0.0407 | 1.98 |
| **HSPD1** | 0.0151 | 1.95 | **CTSD** | 0.043 | 0.50 |
| **CCNB2^#^** | 0.0152 | 1.49 | **TANK** | 0.0438 | 1.27 |
| **BMP5^#^** | 0.0167 | 0.51 | **RAF1** | 0.044 | 0.72 |
| **CSF2** | 0.0169 | 5.30 | **ETS2^#^** | 0.0453 | 1.59 |
| **PCNA** | 0.0175 | 1.62 | **IGF1** | 0.0457 | 0.49 |
| **TOLLIP** | 0.0178 | 0.61 | **GSC** | 0.046 | 2.79 |
| ***BCL2^2^*** | *0.0186* | *0.44* | **BMP4** | 0.0464 | 1.59 |
| **RUNX1** | 0.0188 | 1.85 | **MAP2K7** | 0.0465 | 0.63 |
| **E2F1** | 0.019 | 2.13 | **SLC9A3R1** | 0.0469 | 0.38 |
| **SOCS3** | 0.019 | 5.07 | **NOG** | 0.0472 | 2.02 |
| **LPAR1** | 0.0198 | 0.62 | **BMP1** | 0.0475 | 0.71 |
| **RBBP8** | 0.0198 | 1.59 | **SP1** | 0.0478 | 0.56 |
| **CCNB1** | 0.0199 | 1.79 | **BMP6** | 0.0487 | 0.62 |
| **PPP1R15A** | 0.02 | 1.82 | **DDIT3** | 0.0487 | 1.75 |
| **TNFRSF1A** | 0.0201 | 0.72 | **LDHA** | 0.0489 | 1.53 |
| **WNT5A** | 0.0204 | 2.58 | **PTGDR** | 0.049 | 0.53 |
| **CTBP1** | 0.0206 | 0.55 | **LTBP2** | 0.0491 | 2.83 |

Panel B

| **Gene** | **p Value  (Diabetes vs. Non-Diabetes)** | **Fold Change in Diabetes**** | **Gene** | **p value  (Diabetes vs. Non-Diabetes)** | **Fold Change in Diabetes**** |
| --- | --- | --- | --- | --- | --- |
| ***BCL2^8^*** | *0.0041* | *0.46* | **RELB** | 0.0284 | 1.52 |
| **BMPR1A** | 0.0048 | 0.53 | **TLE1** | 0.0284 | 0.60 |
| **TGFBRAP1** | 0.0064 | 0.68 | ***GUSB^13^*** | *0.0285* | *0.59* |
| **PPP2R1A** | 0.0066 | 0.58 | **APC** | 0.0286 | 0.45 |
| **TMED4** | 0.0067 | 0.77 | **ETS2^#^** | 0.0292 | 0.60 |
| **BMP5^#^** | 0.0069 | 0.44 | **FRZB** | 0.0292 | 0.41 |
| **SMAD3** | 0.0071 | 0.51 | **MAP3K7** | 0.0297 | 0.65 |
| **AKT1** | 0.0072 | 0.64 | **TSC1** | 0.0298 | 0.64 |
| **PTK2** | 0.0079 | 0.64 | **VCAM1** | 0.0298 | 0.54 |
| **SMAD4** | 0.01 | 0.57 | **TGFBR2** | 0.0302 | 0.48 |
| ***YWHAZ^9^*** | *0.0108* | *0.71* | **PIK3R1** | 0.031 | 0.55 |
| **RASA1** | 0.0109 | 0.52 | **ATF2** | 0.0314 | 0.68 |
| **MAPK1** | 0.0116 | 0.63 | **TIRAP** | 0.0317 | 0.72 |
| **NLK** | 0.0121 | 0.70 | **IKBKG** | 0.032 | 0.80 |
| **ITGB5** | 0.0122 | 0.69 | **SMAD5** | 0.0321 | 0.57 |
| **CALM1^#^** | 0.0135 | 0.80 | **JUNB** | 0.0326 | 2.21 |
| **PRKCE** | 0.0141 | 0.52 | **HSPA4** | 0.0329 | 0.64 |
| **PAK1** | 0.0142 | 0.61 | **SENP2** | 0.0336 | 0.69 |
| **SMAD1** | 0.0157 | 0.61 | **FOXO1** | 0.0339 | 0.60 |
| **TP53** | 0.0159 | 0.57 | **BIRC2** | 0.0343 | 0.61 |
| **WASL** | 0.0159 | 0.54 | **PIK3CA** | 0.0362 | 0.58 |
| **S100A6** | 0.0163 | 1.53 | **PIK3R2** | 0.0373 | 0.67 |
| ***MDM2^10^*** | *0.0174* | *0.60* | **CCND2** | 0.0375 | 0.54 |
| **EDN1^#^** | 0.0178 | 0.69 | **CARD4** | 0.0396 | 0.69 |
| **STAT3** | 0.0181 | 0.58 | **FOXA2^#^** | 0.0399 | 0.68 |
| **ATF1** | 0.0184 | 0.68 | **PRKAR1A** | 0.0405 | 0.60 |
| **CDK5** | 0.0196 | 1.64 | **VCL** | 0.0407 | 0.54 |
| **GRB10^#^** | 0.0197 | 0.59 | **NFKB1** | 0.0419 | 0.70 |
| **PRLR** | 0.021 | 0.43 | **TLR5** | 0.0423 | 0.63 |
| **MTOR** | 0.0216 | 0.56 | **FZD5^#^** | 0.0429 | 0.69 |
| ***FAS^11^*** | *0.022* | *0.56* | ***NAIP^14^*** | *0.0436* | *0.63* |
| **MAPK13** | 0.0246 | 0.67 | **STAT5B** | 0.0438 | 0.69 |
| **BCL9** | 0.0256 | 0.59 | **JAK2** | 0.0454 | 0.56 |
| **IFNG** | 0.0258 | 2.79 | **SMURF1** | 0.046 | 0.68 |
| **SRC** | 0.0261 | 0.66 | **PPP3CA** | 0.0461 | 0.64 |
| ***FAS^12^*** | *0.0269* | *0.56* | **TRIM25** | 0.0495 | 0.66 |
| **IL6ST** | 0.0277 | 0.58 | **CCNB2^#^** | 0.0497 | 1.35 |
| **A2M** | 0.0284 | 0.49 |  |  |  |

* Fold change is versus non-tumor samples, ** Fold change is versus non-diabetes samples, ^#^ Significant both in the context of tumor versus non-tumor and diabetic versus non-diabetic groups, ^1^ Assay Hs01005622_m1 (for *FASN*), ^2^ Assay Hs00608023_m1 (for *BCL2*), ^3^ Assay Hs00180269_m1 (for *BAX*), ^4^ Assay Hs03929097_g1 (for *GAPDH*), ^5^ Assay Hs00905030_m1 (for *GAPDH*), ^6^ Assay Hs99999003_m1 (for *MYC*), ^7^ Assay Hs00212390_m1 (for *LEF1*), ^8^ Assay Hs99999018_m (for *BCL2*), ^9^ Assay Hs03044281_g1 (for *YWHAZ*), ^10^ Assay Hs00234753_m1 (for *MDM2*), ^11^ Assay Hs00236330_m1 (for *FAS*), ^12^ Assay Hs00531110_m1 (for *FAS*), ^13^ Assay Hs00939627_m1 (for *GUSB*), ^14^ Assay Hs03037952_m1 (for *NAIP*).

Significantly altered genes were ordered into KEGG (Kyoto Encyclopedia of Genes and Genomes) database categories and terms with the g:Profiler tool. Only terms matching KEGG categories 3 (Environmental Information Processing), 4 (Cellular Processes) or 5 (Organismal Systems) are indicated. Panel A: tumor versus non-tumor; Panel B: diabetic versus non-diabetic.

**Table S2.** KEGG database terms from the enrichment analysis of genes significantly altered either in colon tumor or diabetic samples.

Panel A

| **KEGG Database Main Category** | **KEGG Database Subcategory** | **KEGG Term** | **Adjusted p Value*** | **Query Genes Matching KEGG Term** |
| --- | --- | --- | --- | --- |
| 3. Environmental Information Processing | 3.2 Signal transduction | TGF-beta signaling pathway | 4.33E-09 | BMP2, BMP4, BMP5, BMP6, BMP7, CDKN2B, INHBA, MAPK3, MYC, NOG, SP1, TGFB2, TGFB3 |
| 3. Environmental Information Processing | 3.2 Signal transduction | Hippo signaling pathway | 3.42E-08 | BIRC5, BMP2, BMP4, BMP5, BMP6, BMP7, FZD1, FZD3, FZD5, LEF1, MYC, TGFB2, TGFB3, WNT2, WNT5A |
| 3. Environmental Information Processing | 3.2 Signal transduction | mTOR signaling pathway | 2.69E-06 | FZD1, FZD3, FZD5, GRB10, IGF1, KRAS, MAPK3, RAF1, RHEB, RPS6KA1, TNFRSF1A, WNT2, WNT5A |
| 3. Environmental Information Processing | 3.2 Signal transduction | FoxO signaling pathway | 3.71E-06 | CCNB1, CCNB2, CDKN1A, CDKN2B, GADD45A, IGF1, IL6, KRAS, MAPK3, RAF1, TGFB2, TGFB3 |
| 3. Environmental Information Processing | 3.2 Signal transduction | MAPK signaling pathway | 6.3E-06 | ARRB1, DDIT3, GADD45A, IGF1, KRAS, MAP2K7, MAPK3, MAPK7, MAPK8IP2, MYC, NFKB2, PDGFB, RAF1, RPS6KA1, TGFB2, TGFB3, TNFRSF1A |
| 3. Environmental Information Processing | 3.2 Signal transduction | HIF-1 signaling pathway | 0.000262 | BCL2, CDKN1A, EDN1, GAPDH, HMOX1, IGF1, IL6, LDHA, MAPK3 |
| 3. Environmental Information Processing | 3.2 Signal transduction | PI3K-Akt signaling pathway | 0.000378 | BCL2, BRCA1, CCNE1, CDK4, CDKN1A, GYS1, IGF1, IL6, ITGB7, KRAS, LPAR1, MAPK3, MYC, PDGFB, RAF1, RHEB |
| 3. Environmental Information Processing | 3.2 Signal transduction | TNF signaling pathway | 0.000578 | CSF2, EDN1, IL6, MAP2K7, MAPK3, MMP3, MMP9, SOCS3, TNFRSF1A |
| 3. Environmental Information Processing | 3.3 Signaling molecules and interaction | Cytokine-cytokine receptor interaction | 0.000901 | BMP2, BMP3, BMP4, BMP5, BMP6, BMP7, CSF2, IL10RA, IL6, INHBA, TGFB2, TGFB3, TNFRSF10B, TNFRSF1A |
| 3. Environmental Information Processing | 3.2 Signal transduction | JAK-STAT signaling pathway | 0.002141 | BCL2, CDKN1A, CSF2, IL10RA, IL6,MYC, PDGFB, RAF1, SOCS2, SOCS3 |
| 3. Environmental Information Processing | 3.2 Signal transduction | Wnt signaling pathway | 0.010302 | CTBP1, CXXC4, FZD1, FZD3, FZD5, LEF1, MYC, WNT2, WNT5A |
| 3. Environmental Information Processing | 3.2 Signal transduction | Phospholipase D signaling pathway | 0.033802 | ADCY5, GRM7, KRAS, LPAR1, MAPK3, PDGFB, RAF1, RHEB |
| 3. Environmental Information Processing | 3.2 Signal transduction | ErbB signaling pathway | 0.044041 | CDKN1A, KRAS, MAP2K7, MAPK3, MYC, RAF1 |
| 3. Environmental Information Processing | 3.2 Signal transduction | Sphingolipid signaling pathway | 0.049336 | BAX, BCL2, CTSD, KRAS, MAPK3, RAF1, TNFRSF1A |
| 4. Cellular Processes | 4.2 Cell growth and death | Cellular senescence | 3.28E-11 | CALM1, CCNA2, CCNB1, CCNB2, CCNE1, CDK4, CDKN1A, CDKN2B, E2F1, GADD45A, IL6, KRAS, MAPK3, MYC, RAF1, RHEB, TGFB2, TGFB3 |
| 4. Cellular Processes | 4.2 Cell growth and death | Cell cycle | 2.23E-07 | CCNA2, CCNB1, CCNB2, CCNE1, CDK4, CDKN1A, CDKN2B, E2F1, GADD45A, MYC, PCNA, TGFB2, TGFB3 |
| 4. Cellular Processes | 4.2 Cell growth and death | p53 signaling pathway | 1.1E-06 | BAX, BCL2, CCNB1, CCNB2, CCNE1, CDK4, CDKN1A, GADD45A, IGF1, TNFRSF10B |
| 4. Cellular Processes | 4.3 Cellular community - eukaryotes | Signaling pathways regulating pluripotency of stem cells | 8.55E-06 | BMP4, FZD1, FZD3, FZD5, IGF1, INHBA, KRAS, MAPK3, MYC, RAF1, WNT2, WNT5A |
| 4. Cellular Processes | 4.2 Cell growth and death | Apoptosis | 5.42E-05 | BAX, BCL2, BIRC5, CTSD, DDIT3, GADD45A, KRAS, MAPK3, RAF1, TNFRSF10B, TNFRSF1A |
| 4. Cellular Processes | 4.3 Cellular community - eukaryotes | Gap junction | 0.007609 | ADCY5, KRAS, LPAR1, MAPK3, MAPK7, PDGFB, RAF1 |
| 4. Cellular Processes | 4.2 Cell growth and death | Oocyte meiosis | 0.009383 | ADCY5, CALM1, CCNB1, CCNB2, CCNE1, IGF1, MAPK3, RPS6KA1 |
| 4. Cellular Processes | 4.2 Cell growth and death | Apoptosis - multiple species | 0.047275 | BAX, BCL2, BIRC5, TNFRSF1A |
| 5. Organismal Systems | 5.2 Endocrine system | Melanogenesis | 2.24E-07 | ADCY5, CALM1, EDN1, FZD1, FZD3, FZD5, KRAS, LEF1, MAPK3, RAF1, WNT2, WNT5A |
| 5. Organismal Systems | 5.2 Endocrine system | Parathyroid hormone synthesis, secretion and action | 4.51E-06 | ADCY5, ARRB1, BCL2, BGLAP, CDKN1A, MAPK3, NR4A2, PTH1R, RAF1, SLC9A3R1, SP1 |
| 5. Organismal Systems | 5.6 Nervous system | Neurotrophin signaling pathway | 0.000136 | BAX, BCL2, CALM1, KRAS, MAP2K7, MAPK3, MAPK7, RAF1, RIPK2, RPS6KA1 |
| 5. Organismal Systems | 5.2 Endocrine system | Progesterone-mediated oocyte maturation | 0.000186 | ADCY5, CCNA2, CCNB1, CCNB2, IGF1, KRAS, MAPK3, RAF1, RPS6KA1 |
| 5. Organismal Systems | 5.2 Endocrine system | Insulin signaling pathway | 0.003209 | CALM1, FASN, GYS1, KRAS, MAPK3, RAF1, RHEB, SOCS2, SOCS3 |
| 5. Organismal Systems | 5.2 Endocrine system | Estrogen signaling pathway | 0.0034 | ADCY5, BCL2, CALM1, CTSD, KRAS, MAPK3, MMP9, RAF1, SP1 |
| 5. Organismal Systems | 5.2 Endocrine system | GnRH signaling pathway | 0.010804 | ADCY5, CALM1, KRAS, MAP2K7, MAPK3, MAPK7, RAF1 |
| 5. Organismal Systems | 5.2 Endocrine system | Relaxin signaling pathway | 0.013848 | ADCY5, ARRB1, EDN1, KRAS, MAP2K7, MAPK3, MMP9, RAF1 |

Panel B

| **KEGG Database Main Category** | **KEGG Database Subcategory** | **KEGG Term** | **Adjusted *p* Value*** | **Query Genes Matching KEGG Term** |
| --- | --- | --- | --- | --- |
| 3. Environmental Information Processing | 3.2 Signal transduction | TNF signaling pathway | 1.73E-12 | AKT1, ATF2, BIRC2, EDN1, FAS, IKBKG, JUNB, MAP3K7, MAPK1, MAPK13, NFKB1, PIK3CA, PIK3R1, PIK3R2, VCAM1 |
| 3. Environmental Information Processing | 3.2 Signal transduction | FoxO signaling pathway | 1.93E-11 | AKT1, CCNB2, CCND2, FOXO1, MAPK1, MAPK13, MDM2, NLK, PIK3CA, PIK3R1, PIK3R2, SMAD3, SMAD4, STAT3, TGFBR2 |
| 3. Environmental Information Processing | 3.2 Signal transduction | PI3K-Akt signaling pathway | 9.36E-10 | AKT1, ATF2, BCL2, CCND2, IKBKG, ITGB5, JAK2, MAPK1, MDM2, MTOR, NFKB1, PIK3CA, PIK3R1, PIK3R2, PPP2R1A, PRLR, PTK2, TP53, TSC1, YWHAZ |
| 3. Environmental Information Processing | 3.2 Signal transduction | TGF-beta signaling pathway | 2.62E-08 | BMP5, BMPR1A, IFNG, MAPK1, PPP2R1A, SMAD1, SMAD3, SMAD4, SMAD5, SMURF1, TGFBR2 |
| 3. Environmental Information Processing | 3.2 Signal transduction | HIF-1 signaling pathway | 7.38E-08 | AKT1, BCL2, EDN1, IFNG, MAPK1, MTOR, NFKB1, PIK3CA, PIK3R1, PIK3R2, STAT3 |
| 3. Environmental Information Processing | 3.2 Signal transduction | JAK-STAT signaling pathway | 1E-07 | AKT1, BCL2, CCND2, IFNG, IL6ST, JAK2, MTOR, PIK3CA, PIK3R1, PIK3R2, PRLR, STAT3, STAT5B |
| 3. Environmental Information Processing | 3.2 Signal transduction | VEGF signaling pathway | 1.44E-07 | AKT1, MAPK1, MAPK13, PIK3CA, PIK3R1, PIK3R2, PPP3CA, PTK2, SRC |
| 3. Environmental Information Processing | 3.2 Signal transduction | ErbB signaling pathway | 2.08E-07 | AKT1, MAPK1, MTOR, PAK1, PIK3CA, PIK3R1, PIK3R2, PTK2, SRC, STAT5B |
| 3. Environmental Information Processing | 3.2 Signal transduction | Sphingolipid signaling pathway | 4.81E-07 | AKT1, BCL2, MAPK1, MAPK13, NFKB1, PIK3CA, PIK3R1, PIK3R2, PPP2R1A, PRKCE, TP53 |
| 3. Environmental Information Processing | 3.2 Signal transduction | Hippo signaling pathway | 6.53E-07 | APC, BIRC2, BMP5, BMPR1A, CCND2, FZD5, PPP2R1A, SMAD1, SMAD3, SMAD4, TGFBR2, YWHAZ |
| 3. Environmental Information Processing | 3.2 Signal transduction | MAPK signaling pathway | 2.42E-06 | AKT1, ATF2, FAS, IKBKG, MAP3K7, MAPK1, MAPK13, NFKB1, NLK, PAK1, PPP3CA, RASA1, RELB, TGFBR2, TP53 |
| 3. Environmental Information Processing | 3.2 Signal transduction | NF-kappa B signaling pathway | 8.7E-06 | BCL2, BIRC2, IKBKG, MAP3K7, NFKB1, RELB, TIRAP, TRIM25, VCAM1 |
| 3. Environmental Information Processing | 3.2 Signal transduction | AMPK signaling pathway | 7.28E-05 | AKT1, FOXO1, MAP3K7, MTOR, PIK3CA, PIK3R1, PIK3R2, PPP2R1A, TSC1 |
| 3. Environmental Information Processing | 3.2 Signal transduction | Wnt signaling pathway | 8.98E-05 | APC, CCND2, FZD5, MAP3K7, NLK, PPP3CA, SENP2, SMAD3, SMAD4, TP53 |
| 3. Environmental Information Processing | 3.2 Signal transduction | Ras signaling pathway | 0.000394 | AKT1, CALM1, ETS2, IKBKG, MAPK1, NFKB1, PAK1, PIK3CA, PIK3R1, PIK3R2, RASA1 |
| 3. Environmental Information Processing | 3.2 Signal transduction | mTOR signaling pathway | 0.00056 | AKT1, FZD5, GRB10, MAPK1, MTOR, PIK3CA, PIK3R1, PIK3R2, TSC1 |
| 3. Environmental Information Processing | 3.2 Signal transduction | cAMP signaling pathway | 0.007847 | AKT1, CALM1, EDN1, MAPK1, NFKB1, PAK1, PIK3CA, PIK3R1, PIK3R2 |
| 3. Environmental Information Processing | 3.2 Signal transduction | Apelin signaling pathway | 0.015907 | AKT1, CALM1, MAPK1, MTOR, PRKCE, SMAD3, SMAD4 |
| 3. Environmental Information Processing | 3.2 Signal transduction | Phospholipase D signaling pathway | 0.024542 | AKT1, MAPK1, MTOR, PIK3CA, PIK3R1, PIK3R2, TSC1 |
| 3. Environmental Information Processing | 3.2 Signal transduction | Rap1 signaling pathway | 0.035912 | AKT1, CALM1, MAPK1, MAPK13, PIK3CA, PIK3R1, PIK3R2, SRC |
| 4. Cellular Processes | 4.2 Cell growth and death | Cellular senescence | 7.86E-14 | AKT1, CALM1, CCNB2, CCND2, FOXO1, MAPK1, MAPK13, MDM2, MTOR, NFKB1, PIK3CA, PIK3R1, PIK3R2, PPP3CA, SMAD3, TGFBR2, TP53, TSC1 |
| 4. Cellular Processes | 4.3 Cellular community - eukaryotes | Signaling pathways regulating pluripotency of stem cells | 3.26E-12 | AKT1, APC, BMPR1A, FZD5, IL6ST, JAK2, MAPK1, MAPK13, PIK3CA, PIK3R1, PIK3R2, SMAD1, SMAD3, SMAD4, SMAD5, STAT3 |
| 4. Cellular Processes | 4.3 Cellular community - eukaryotes | Adherens junction | 8.92E-07 | MAP3K7, MAPK1, NLK, SMAD3, SMAD4, SRC, TGFBR2, VCL, WASL |
| 4. Cellular Processes | 4.3 Cellular community - eukaryotes | Focal adhesion | 1.17E-06 | AKT1, BCL2, BIRC2, CCND2, ITGB5, MAPK1, PAK1, PIK3CA, PIK3R1, PIK3R2, PTK2, SRC, VCL |
| 4. Cellular Processes | 4.2 Cell growth and death | Apoptosis | 1.83E-06 | AKT1, BCL2, BIRC2, FAS, IKBKG, MAPK1, NFKB1, PIK3CA, PIK3R1, PIK3R2, TP53 |
| 4. Cellular Processes | 4.1 Transport and catabolism | Autophagy - animal | 0.000126 | AKT1, BCL2, MAP3K7, MAPK1, MTOR, PIK3CA, PIK3R1, PIK3R2, TSC1 |
| 4. Cellular Processes | 4.5 Cell motility | Regulation of actin cytoskeleton | 0.000179 | APC, ITGB5, MAPK1, PAK1, PIK3CA, PIK3R1, PIK3R2, PTK2, SRC, VCL, WASL |
| 4. Cellular Processes | 4.2 Cell growth and death | p53 signaling pathway | 0.003122 | BCL2, CCNB2, CCND2, FAS, MDM2, TP53 |
| 4. Cellular Processes | 4.2 Cell growth and death | Cell cycle | 0.00853 | CCNB2, CCND2, MDM2, SMAD3, SMAD4, TP53, YWHAZ |
| 4. Cellular Processes | 4.2 Cell growth and death | Necroptosis | 0.044204 | BCL2, BIRC2, FAS, IFNG, JAK2, STAT3, STAT5B |
| 5. Organismal Systems | 5.2 Endocrine system | Prolactin signaling pathway | 1.72E-12 | AKT1, CCND2, JAK2, MAPK1, MAPK13, NFKB1, PIK3CA, PIK3R1, PIK3R2, PRLR, SRC, STAT3, STAT5B |
| 5. Organismal Systems | 5.1 Immune system | C-type lectin receptor signaling pathway | 1.68E-11 | AKT1, CALM1, IKBKG, MAPK1, MAPK13, MDM2, NFKB1, PAK1, PIK3CA, PIK3R1, PIK3R2, PPP3CA, RELB, SRC |
| 5. Organismal Systems | 5.1 Immune system | Th17 cell differentiation | 1.68E-11 | IFNG, IKBKG, IL6ST, JAK2, MAPK1, MAPK13, MTOR, NFKB1, PPP3CA, SMAD3, SMAD4, STAT3, STAT5B, TGFBR2 |
| 5. Organismal Systems | 5.8 Development and regeneration | Osteoclast differentiation | 2.25E-10 | AKT1, IFNG, IKBKG, JUNB, MAP3K7, MAPK1, MAPK13, NFKB1, PIK3CA, PIK3R1, PIK3R2, PPP3CA, RELB, TGFBR2 |
| 5. Organismal Systems | 5.1 Immune system | T cell receptor signaling pathway | 4.16E-09 | AKT1, IFNG, IKBKG, MAP3K7, MAPK1, MAPK13, NFKB1, PAK1, PIK3CA, PIK3R1, PIK3R2, PPP3CA |
| 5. Organismal Systems | 5.1 Immune system | Chemokine signaling pathway | 5.78E-08 | AKT1, IKBKG, JAK2, MAPK1, NFKB1, PAK1, PIK3CA, PIK3R1, PIK3R2, PTK2, SRC, STAT3, STAT5B, WASL |
| 5. Organismal Systems | 5.2 Endocrine system | Relaxin signaling pathway | 8.48E-08 | AKT1, ATF2, EDN1, MAPK1, MAPK13, NFKB1, PIK3CA, PIK3R1, PIK3R2, SMAD3, SRC, TGFBR2 |
| 5. Organismal Systems | 5.1 Immune system | Toll-like receptor signaling pathway | 9.15E-08 | AKT1, IKBKG, MAP3K7, MAPK1, MAPK13, NFKB1, PIK3CA, PIK3R1, PIK3R2, TIRAP, TLR5 |
| 5. Organismal Systems | 5.9 Aging | Longevity regulating pathway | 2.96E-07 | AKT1, ATF2, FOXO1, MTOR, NFKB1, PIK3CA, PIK3R1, PIK3R2, TP53, TSC1 |
| 5. Organismal Systems | 5.2 Endocrine system | Thyroid hormone signaling pathway | 4.92E-06 | AKT1, FOXO1, MAPK1, MDM2, MTOR, PIK3CA, PIK3R1, PIK3R2, SRC, TP53 |
| 5. Organismal Systems | 5.6 Nervous system | Neurotrophin signaling pathway | 6.29E-06 | AKT1, BCL2, CALM1, MAPK1, MAPK13, NFKB1, PIK3CA, PIK3R1, PIK3R2, TP53 |
| 5. Organismal Systems | 5.1 Immune system | B cell receptor signaling pathway | 1.26E-05 | AKT1, IKBKG, MAPK1, NFKB1, PIK3CA, PIK3R1, PIK3R2, PPP3CA |
| 5. Organismal Systems | 5.2 Endocrine system | Insulin signaling pathway | 2.23E-05 | AKT1, CALM1, FOXO1, MAPK1, MTOR, PIK3CA, PIK3R1, PIK3R2, PRKAR1A, TSC1 |
| 5. Organismal Systems | 5.9 Aging | Longevity regulating pathway - multiple species | 6.86E-05 | AKT1, FOXA2, FOXO1, MTOR, PIK3CA, PIK3R1, PIK3R2 |
| 5. Organismal Systems | 5.1 Immune system | Th1 and Th2 cell differentiation | 8.26E-05 | IFNG, IKBKG, JAK2, MAPK1, MAPK13, NFKB1, PPP3CA, STAT5B |
| 5. Organismal Systems | 5.1 Immune system | Fc gamma R-mediated phagocytosis | 9E-05 | AKT1, MAPK1, PAK1, PIK3CA, PIK3R1, PIK3R2, PRKCE, WASL |
| 5. Organismal Systems | 5.2 Endocrine system | Estrogen signaling pathway | 0.000238 | AKT1, ATF2, BCL2, CALM1, MAPK1, PIK3CA, PIK3R1, PIK3R2, SRC |
| 5. Organismal Systems | 5.8 Development and regeneration | Axon guidance | 0.000294 | CDK5, MAPK1, PAK1, PIK3CA, PIK3R1, PIK3R2, PPP3CA, PTK2, RASA1, SRC |
| 5. Organismal Systems | 5.1 Immune system | Natural killer cell mediated cytotoxicity | 0.000947 | FAS, IFNG, MAPK1, PAK1, PIK3CA, PIK3R1, PIK3R2, PPP3CA |
| 5. Organismal Systems | 5.1 Immune system | NOD-like receptor signaling pathway | 0.001594 | BCL2, BIRC2, CARD4, IKBKG, MAP3K7, MAPK1, MAPK13, NAIP, NFKB1 |
| 5. Organismal Systems | 5.2 Endocrine system | Progesterone-mediated oocyte maturation | 0.001644 | AKT1, CCNB2, MAPK1, MAPK13, PIK3CA, PIK3R1, PIK3R2 |
| 5. Organismal Systems | 5.1 Immune system | Fc epsilon RI signaling pathway | 0.002065 | AKT1, MAPK1, MAPK13, PIK3CA, PIK3R1, PIK3R2 |
| 5. Organismal Systems | 5.7 Sensory system | Inflammatory mediator regulation of TRP channels | 0.002147 | CALM1, MAPK13, PIK3CA, PIK3R1, PIK3R2, PRKCE, SRC |
| 5. Organismal Systems | 5.2 Endocrine system | Adipocytokine signaling pathway | 0.002249 | AKT1, IKBKG, JAK2, MTOR, NFKB1, STAT3 |
| 5. Organismal Systems | 5.1 Immune system | Leukocyte transendothelial migration | 0.004467 | MAPK13, PIK3CA, PIK3R1, PIK3R2, PTK2, VCAM1, VCL |
| 5. Organismal Systems | 5.6 Nervous system | Cholinergic synapse | 0.004467 | AKT1, BCL2, JAK2, MAPK1, PIK3CA, PIK3R1, PIK3R2 |
| 5. Organismal Systems | 5.1 Immune system | Platelet activation | 0.00853 | AKT1, MAPK1, MAPK13, PIK3CA, PIK3R1, PIK3R2, SRC |
| 5. Organismal Systems | 5.1 Immune system | IL-17 signaling pathway | 0.011664 | IFNG, IKBKG, MAP3K7, MAPK1, MAPK13, NFKB1 |
| 5. Organismal Systems | 5.3 Circulatory system | Adrenergic signaling in cardiomyocytes | 0.022568 | AKT1, ATF2, BCL2, CALM1, MAPK1, MAPK13, PPP2R1A |
| 5. Organismal Systems | 5.5 Excretory system | Aldosterone-regulated sodium reabsorption | 0.024155 | MAPK1, PIK3CA, PIK3R1, PIK3R2 |
| 5. Organismal Systems | 5.1 Immune system | RIG-I-like receptor signaling pathway | 0.029641 | IKBKG, MAP3K7, MAPK13, NFKB1, TRIM25 |
| 5. Organismal Systems | 5.4 Digestive system | Carbohydrate digestion and absorption | 0.047375 | AKT1, PIK3CA, PIK3R1, PIK3R2 |

*Adjustment for multiple testing was carried out with the g:SCS algorithm of g:Profiler, suitable for analysis of hierarchically related terms.
