# Supplementary figures and images for: Correlation between Expression Profiles of Key Signaling Genes in Colorectal Cancer Samples from Type 2 Diabetic and Non-Diabetic Patients
Source: Life (Basel). 2020 Sep 22;10(9):216. doi: 10.3390/life10090216 (PMC7555724; doi:10.3390/life10090216)

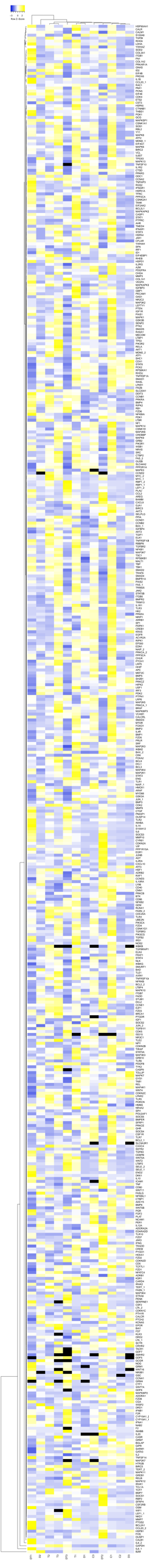

Supplement: Supplementary file 1 [file life-10-00216-s001.zip › life-892598-supplementary- for XML/Supplementary Figure 1.pdf]

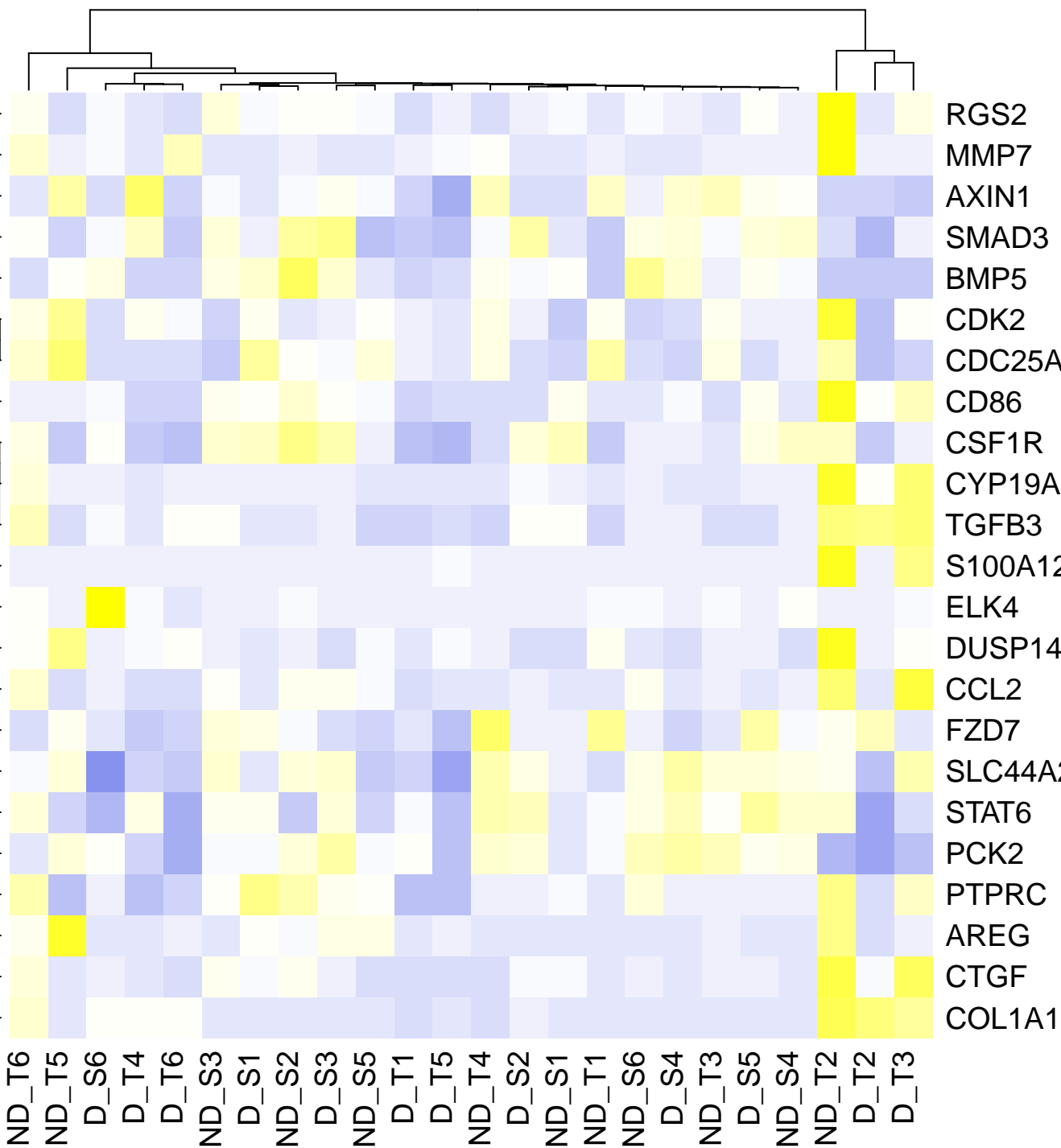

Supplement: Supplementary file 1 [file life-10-00216-s001.zip › life-892598-supplementary- for XML/Supplementary Figure 2.pdf]
